# The central nucleus of the amygdala lesion attenuates orthodontic pain during experimental tooth movement in rats

**DOI:** 10.1002/brb3.1506

**Published:** 2019-12-20

**Authors:** Hu Qiao, Yunan Gao, Qianqian Huang, Ru Jia

**Affiliations:** ^1^ Key Laboratory of Shaanxi Province for Craniofacial Precision Medicine Research Xi'an Jiaotong University College of Stomatology Xi'an Shaanxi China; ^2^ Department of Orthodontics Xi'an Jiaotong University College of Stomatology Xi'an Shaanxi China

**Keywords:** orthodontic pain, the central nucleus of the amygdala, tooth movement

## Abstract

**Introduction:**

Orthodontic pain is the most common adverse side effect reported in the context of tooth movement. Given its central role in processing pain and negative emotion, the central nucleus of the amygdala (CeA) is thought to be a key site involved in orthodontic pain sensation.

**Methods:**

In the present study, we therefore explored whether the CeA is involved in contributing to orthodontic pain in a rat model of tooth movement. For this study, we utilized adult male rats with bilateral sham or electrolytic CeA lesions (400 μA; 25 s), and then we analyzed face grooming behavior as a measure of pain sensation.

**Results:**

Through this approach, we found that there were time‐ and force‐dependent factors influencing pain levels in these rats. We further found that bilateral CeA lesions markedly reduced tooth movement‐induced orofacial pain and that unilateral CeA lesions did so to a lesser extent.

**Conclusions:**

As such, these results suggest the CeA is a key area of orthodontic pain, with the results of this study highlighting potential avenues for achieving pain relief in those suffering from orthodontic pain.

## Graphical abstract

We found that bilateral CeA lesions markedly reduced tooth movement‐induced orofacial pain and that unilateral CeA lesions did so to a lesser extent. These results suggest the CeA is a key area of orthodontic pain, with the results of this study highlighting potential avenues for achieving pain relief in those suffering from orthodontic pain.

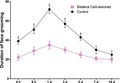

## 1 INTRODUCTION

Tooth movement‐associated pain can be detected throughout orthodontic treatment (Banerjee, Banerjee, Shenoy, Agarkar, & Bhattacharya, 2018), with up to 85% of patient suffering mild‐to‐moderate pain, and up to 9% of patients suffering from severe pain on the initial day of the treatment process (Campos, Fraga, Raposo, Ferreira, & Vitral, 2013). Such pain is one of the primary reasons why patients terminate or otherwise fail to comply with treatment regimens (Bergius, Berggren, & Kiliaridis, 2002; Krishnan, 2007). As such, it is vital that clinical methods for managing such orthodontic pain be developed.

Currently, the majority of studies focus on peripheral mechanisms governing orthodontic pain, with relatively few reports regarding the central mechanisms. Pain sensation is governed by several cerebral structures that comprise the pain matrix, and which are involved in detecting, expressing, and modulating pain. These structures are capable of transmitting nociceptive information and are thereby able to reduce or enhance pain sensation to alter patient affect and to promote defensive or distressed behaviors. Of the structures involved in such sensation, the amygdala in particular is known to be a key player in pain sensation (Simons et al., 2014).

In response to pain, amygdala activation is evident in both rodents and humans (Carrasquillo & Gereau, 2007; Ikeda, Takahashi, Inoue, & Kato, 2007). The role for this structure in modulating pain sensation is well‐supported by many different anatomical, behavioral, and physiological research efforts (Berman et al., 2006; Mayer et al., 2005; Naliboff et al., 2003). The central nucleus of the amygdala (CeA) is a structure that is ideally positioned to process nociceptive inputs from the spino‐parabrachio‐amygdaloid pathway (Neugebauer, Li, Bird, & Han, 2004), transmitting the resultant information to hypothalamus, substantia inominata dorsalis, and nuclei within the brainstem controlling defensive behavioral responses (Aggleton, Vann, Oswald, & Good, 2000). While these reports have shown the CeA to be essential for antinociceptive activity in model systems, its specific function in orthodontic pain sensation remains uncertain.

In accordance with theories of pain and the known roles of the CeA, we hypothesized that the CeA drives orthodontic pain. In this study, we therefore explored how CeA lesions affected orthodontic pain development.

## 2 METHODS

### 2.1 Animals

Male Sprague Dawley rats (200–250 g) were from the Medical Experimental Animal Center of the Xi'an Jiaotong University (Xi'an, China) and were housed in standard cages with free food/water access, a 12 hr light/dark cycle, and a temperature‐controlled (18–20°C) environment. Animals were given 5 days to acclimate before experimental use.

The NIH Guide for the Care and Use of Laboratory Animals was observed when planning animal studies, which received approval from the Institutional Animal Care Committee of Xi'an Jiaotong University. Animal numbers and pain were minimized wherever possible, based on animal research ethical guidelines (Zimmermann, 1983).

### 2.2 Experimental tooth movement

The mesial movement of the left maxillary first molar was achieved using a fixed, nickel titanium alloy closed‐coil spring device, as discussed in previous reports (Qiao, Gao, Zhang, & Zhou, 2015). This device contained a closed‐coil spring hooked between a small metal plate attached to the maxillary first molar and a hook attached to a band cemented on the upper incisor.

### 2.3 Behavior test

Previous work has shown that a range of different self‐directed face grooming behaviors in rats reliably allow for the measurement of pain in models of experimental tooth movement (Yang, Luo, et al., 2009). Such behaviors include shaking of the head, paw licking, ear grasping, and chin/mouth wiping. As such, we placed animals into transparent cages (30 cm × 30 cm × 30 cm) in a room with 45‐dB of background noise for 3 hr, beginning to record mouth wiping behavior after a 15‐min acclimatization period. Such behaviors were monitored in each animal for 10 min per time point, with three measurements made 20 min apart. An investigator blinded to experimental protocols then analyzed rats for mouth wiping behavior, with mean values for each animal determined based on the average of the three measured time points.

### 2.4 Electrolytic CeA lesions

Animals were first anesthetized using isoflurane (5%) in 30% oxygen (O_2_) and 70% nitrous oxide (N_2_O). Animals were then transferred to a stereotaxic instrument (SR‐6N; Narishige Scientific Instrument Lab), with the skull being opened at the level between the bregma and lambda. At all time points during surgery, animals were administered 2%–3% isoflurane via a facial mask.

Bilateral CeA lesions were generated via conducting two small craniotomys just over the amygdala. A concentric electrode (CEA 200; MicroProbes) connected to the Lesion‐Making Device (53500; Ugo Basile) was inserted into the brain 2.4 mm caudal to bregma, 4.1 mm from the midline, and 7.5 mm in depth from the cortical surface (Paxinos & Watson, 2005). A constant current (400 µA; for 25 s) was used to generate lesions at the indicated points, with a clip attached to the tail serving as an indifferent electrode. For sham lesion animals, the same surgery and electrode positioning was conducted, but no current was administered to animals. Animals were given 3 days to recover from this operation.

### 2.5 Experimental protocol

Animals were anesthetized as above, after which 30, 50, or 80 cN force was applied to the teeth on one side of rats and the behavior were recorded at 4 hr, 8 hr, 1 day, 3 days, 5 days, 7 days, and 14 days. Sham animals underwent the same operative procedures, but springs were left inactive. Changes in behavioral responses over time were monitored in rats (*n* = 8/group).

In certain experiments, rats that had undergone CeA or sham lesion surgeries were used in this same model of tooth movement‐associated pain. For these animals, 50 cN force was applied for 4 hr, 8 hr, 1 day, 3 days, 5 days, 7 days, and 14 days, with animals being monitored at the indicated time points (*n* = 8/group).

### 2.6 Histology

**Figure 1:**
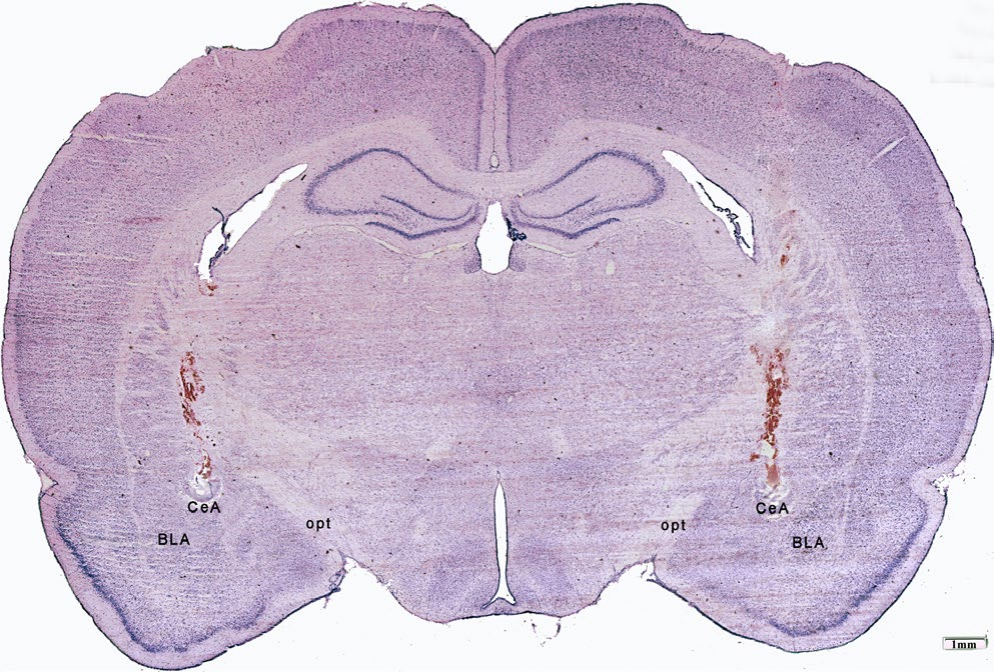
Coronal images of typical bilateral electrolytic CeA lesions. BLA, basolateral amygdala; CeA, central nucleus of amygdala; opt, optic tract. Scale bar = 1 mm

Following the completion of behavioral testing, pentobarbital sodium (50 mg/kg, i.p.) was used to anesthetize animals, followed by perfusion using a 200 ml normal saline and 400 ml 4% paraformaldehyde delivered through the aorta. Samples of brain tissue were submerged in 4% paraformaldehyde overnight, after which they were incubated in 30% sucrose in PBS (pH 7.2) for an additional 24 hr. Next, 30 mm coronal brain sections were prepared and subjected to Nissl staining in order to confirm amygdala lesion location under microscopy. CeA damage is illustrated in Figure 1.

### 2.7 Statistical analysis

Data are means ± *SEM*. Two‐way mixed model ANOVAs were used to assess differences between groups over time. When a significant interaction was detected, directed face grooming was compared between groups via the Bonferonni post hoc test. *p* < .05 was the significance threshold.

## 3 RESULTS

### 3.1 Tissue histology

Typical bilateral CeA lesion sites are shown in Figure 1. Of the 41 animals in which lesions were generated, 9 were rejected as their lesions were either too large and/or they encroached on other portions of the amygdala too significantly. As such, a total of 16 lesioned and 16 sham control animals were used for statistical analyses.

### 3.2 Normal rat pain responses to tooth movement

Firstly, we want to know the normal rat pain responses caused by tooth movement and the animal model was established. We found that in normal rats, tooth movement was associated with a significant increase in directed face grooming at 4 hr–7 days (*p* < .05) relative to sham control animals. Maximal face grooming was evident on day 1 and reduced slowly until it no longer differed from that in control animals at day 14.

**Figure 2:**
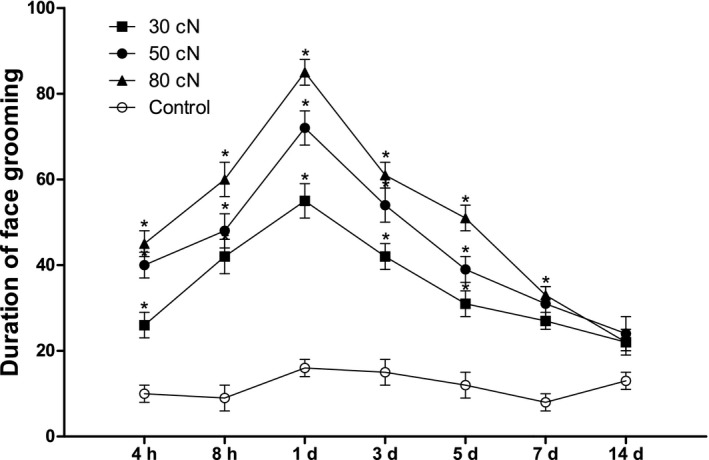
Quantification of rat directed face grooming in response to experimental tooth movement (*Significant difference compared with control group). *p* < .05 = significant difference; results are expressed as means ± *SEM*. *N* = 8 rats/group

We further explored the impact of applying different amounts of force on face grooming behavior, revealing that significantly higher amounts of force significantly increased directed face grooming, with significantly different time course curves between treatment groups [*F*(3, 28) = 30.64, *p* < .05)] (Figure 2).

### 3.3 CeA lesioned rat pain responses to tooth movement

**Figure 3:**
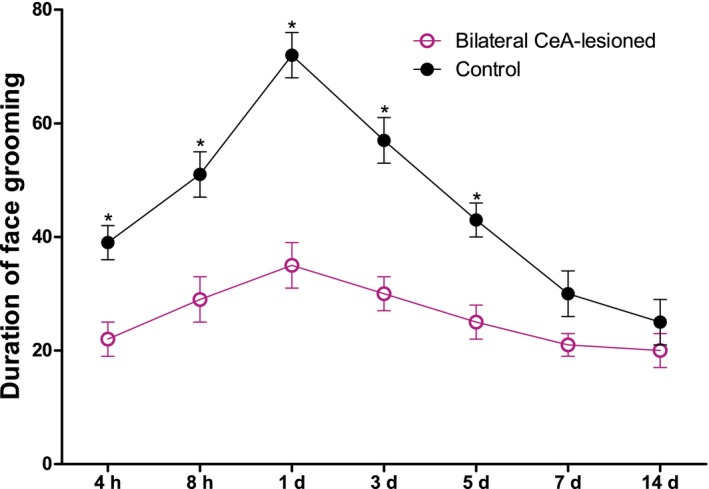
Tooth movement‐induced directed face grooming in bilateral CeA lesion or sham rats. (*Significant difference compared with control group). *p* < .05 = significant difference; results are expressed as means ± *SEM*. *N* = 8 rats/group

**Figure 4:**
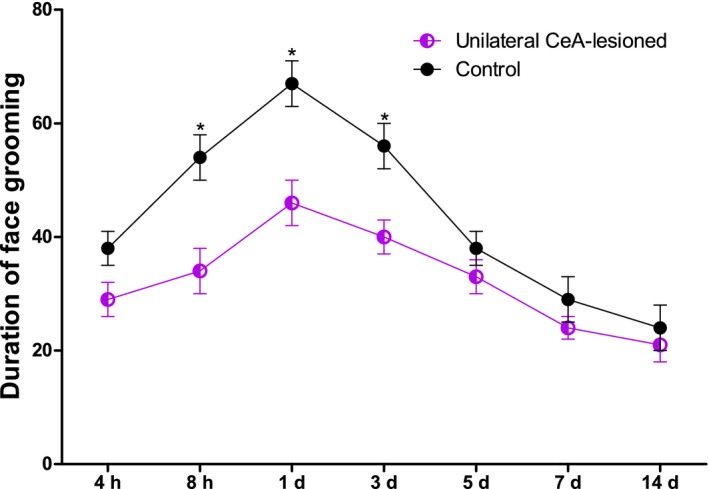
Tooth movement‐induced directed face grooming in unilateral CeA lesion or sham rats. (*Significant difference compared with control group). *p* < .05 = significant difference; results are expressed as means ± *SEM*. *N* = 8 rats/group

Next, we study whether the CeA is involved in the orthodontic pain induced by tooth movement. To address this question, the bilateral or unilateral CeA had been lesioned and the behavior was tested. We found that bilateral CeA lesions induced 3 days prior were associated with a marked reduction in tooth movement‐induced face grooming at 4 hr–5 days (*p* < .05) (Figure 3). In contrast, unilateral CeA lesions only reduced tooth movement‐induced directed face grooming at 8 hr–3 days (*p* < .05), with no significant differences at 4 hr, 5 days, 7 days, or 14 days (*p* > .05). This reduction was markedly reduced relative to that mediated by bilateral CeA lesions at 4 hr–5 days (Figure 4).

## 4 DISCUSSION

Most patients undergoing orthodontic treatments suffer some amount of pain or discomfort during the tooth movement process (Ashkenazi, Berlin‐Broner, & Levin, 2012), with such pain being the most common reason for patients to discontinue treatment. Such pain is typically felt within 4 hr, before peaking after 1 day and gradually abating over the following 5–7 days (Bondemark, Fredriksson, & Ilros, 2004).

While a number of studies have explored orthodontic pain, the central pain pathways that are induced as a consequence of tooth movement are not well‐characterized (Topolski, Moro, Correr, & Schimim, 2018). To explore these pathways in depth, we therefore used a rat model of experimental tooth movement, using directed face grooming behaviors in order to reliably measure pain in these animals, as has been validated previously (Yang, Cao, et al., 2009). Using this approach, we were able to confirm that there was an increase in face grooming behavior as tooth movement force was increased, confirming that these behavioral changes were directly associated with the orthodontic pain in these animals. This pain is perceptible within 2 hr, before peaking after 1 day and gradually declining in the following days (Almasoud, 2018). As we observed significant increases in this behavior relative to baseline, this confirmed that tooth movement was inducing a pain response in these animals.

The amygdala is a complex structure that is situated bilaterally deep within the temporal lobe. It is made up of several regions that are networked together, with each region having unique neurochemical features, connectivity, and architecture (Knapska, Radwanska, Werka, & Kaczmarek, 2007). The amygdala is centrally located, and thus. it is well‐connected to many regions of the brain (Price, 2003), suggesting it can contribute both to the emotional and the cognitive aspects of pain, such as pain‐associated memories and expectations. The amygdala is of particular importance for nociceptive signaling, with multiple preclinical (Apkarian et al., 2013; Neugebauer, 2015) and clinical (Simons et al., 2014; Vachon‐Presseau et al., 2016) studies having directly confirmed an association between the amygdala and pain.

Research suggests the amygdala is involved in both facilitating and inhibiting pain (Manning, Merin, Meng, & Amaral, 2001; Tershner & Helmstetter, 2000). This may be because the amygdala is directly connected to regions of the brainstem associated with pro‐ and antinociception (Almeida, Storkson, Lima, Hole, & Tjolsen, 1999; Porreca, Ossipov, & Gebhart, 2002). The amygdala is also particularly important for the emotional‐affective facets of perception of pain (Veinante, Yalcin, & Barrot, 2013). The CeA in particular has been referred to as the “nociceptive amygdala” owing to of its ability to integrate multiple nociceptive inputs from the thalamus and brainstem together with emotional information as it pertains to pain. This CeA region therefore processes direct and indirect nociceptive inputs. Consistent with this, previous work has shown that experimental tooth movement can induce thalamic and hypothalamic Fos expression, in additional to central amygdala expression (Novaes, Rocha, & Leite‐Panissi, 2010; Yamashiro et al., 1998). As such, we hypothesized that the CeA was likely linked to orthodontic pain.

Our findings indicated that bilateral CeA lesions were able to markedly reduce orthodontic pain in rats, suggesting that the CeA is essential for facilitating tooth movement‐associated pain. We further found that unilateral CeA lesions similarly reduced orthodontic pain, albeit to a lesser extent relative to bilateral lesions, with unilateral lesions only somewhat reducing directed face grooming behavior. In addition, whereas bilateral lesions were able to reduce directed grooming behavior for the duration of testing, unilateral lesions only did so for the period 8 hr–3 days after initiation of tooth movement, failing to do so at 4 hr, and 5 days, likely due to the reduced extent of the lesions in these animals. While this suggests that unilateral CeA lesions can influence behavior associated with orthodontic pain, it is necessary to consider that all the rats included in the group of unilateral CeA lesion were selected from the rats that had bilateral lesions: in the CeA in one side and more or less close to CeA in the other side (that might be also in another nucleus of the amygdala). As such, further research on rats with unilateral CeA lesions will be needed to definitively determine how such unilateral lesions influence tooth movement‐induced directed face grooming.

Our results provide interesting preliminary evidence extending the role of the CeA to orthodontic pain induced in response to tooth movement, although further exploration of the underlying molecular mechanisms is still needed. While facial grooming is the most reliable means of assessing orthodontic pain in rats, how CeA lesions may affect other manifestations of pain in rats still warrants further research, as does how such lesions affect other orthodontic pain models. Additional research into how such pain processing in the CeA interacts with negative emotions is also warranted.

In summary, in the present study we describe a novel mechanism of orthodontic pain sensation, with uni‐ or bilateral CeA lesions reducing such pain in a rat model system. This suggests that the activity of the CeA following tooth movement is important for controlling the development of orthodontic pain. These findings further suggest that targeting the amygdala may be a viable strategy for reducing pain in those undergoing orthodontic procedure and suffering from pain associated with tooth movement.

## CONFLICT OF INTEREST

All the authors listed above declared no conflict of interest.

## AUTHOR CONTRIBUTIONS

All authors had full access to all the data in the study and take responsibility for the integrity of the data and the accuracy of the data analysis; H.Q., Y.N.G., and R.J involved in Conceptualization; H.Q., Y.N.G., and Q.Q.H. involved in Methodology, Formal Analysis, Writing—Original Draft, and Writing—Review & Editing.

## Data Availability

The data that support the findings of this study are available from the corresponding author upon reasonable request.
